# AidData’s Geospatial Global Chinese Development Finance Dataset

**DOI:** 10.1038/s41597-024-03341-w

**Published:** 2024-06-11

**Authors:** Seth Goodman, Sheng Zhang, Ammar A. Malik, Bradley C. Parks, Jacob Hall

**Affiliations:** AidData, William & Mary, Williamsburg, Virginia USA

**Keywords:** Developing world, Geography

## Abstract

AidData’s Global Chinese Development Finance Dataset (Version 3.0) provides detailed information about more than 20,000 development projects across 165 low- and middle-income countries financed by 791 official sector Chinese donors and lenders from 2000 to 2021. In this study, we introduce a methodology for identifying the geospatial features of these projects. Our application of the methodology has resulted in the Geospatial Global Chinese Development Finance Dataset (Version 3.0), which captures the geospatial features of 9,405 projects across 148 low- and middle-income countries supported by Chinese grant and loan commitments worth more than USD 830 billion. The dataset provides details for 6,266 projects containing spatial definitions of roads, railways, power plants, transmission lines, buildings, and other precisely geocoded features. It identifies approximate and administrative-level locations for 3,139 additional projects. The methodology, dataset, and the code used to construct the dataset have been made publicly available to facilitate replication and future applications.

## Background & Summary

Through its flagship overseas infrastructure development program, the Belt and Road Initiative, China has emerged as the single largest official source of aid and credit to low-income and middle-income countries^1^. Between 2000 and 2021, it issued grant and loan commitments worth $1.34 trillion for 20,985 projects across 165 low- and middle-income countries^2,3^. China is now outspending the United States, the World Bank, and all other bilateral and multilateral sources of international development finance^1^.

Previous research suggests that China’s overseas project portfolio has effectively promoted economic growth and development^4–10^. However, a common criticism is that its projects lack adequate environmental, social, and governance (ESG) safeguards^11,12^. A separate but related criticism is that China has effectively prioritized speed and scale over safety^1^. While there is some evidence to support these claims, the empirical study of international development finance from China is rapidly evolving as scholars from the social sciences and the natural sciences seek to overcome an array of informational obstacles^2,9,13–18^. Chief among these obstacles is the fact that Beijing shrouds its overseas lending and grant-giving program in secrecy^19^.

Over the last decade, several university research centers and think tanks have used publicly available sources to assemble datasets—including, but not limited to, China’s Overseas Development Finance Database, the Chinese Loans to Latin America and the Caribbean Database, the Chinese Loans to Africa Database, the China’s Global Energy Finance Database, and the China Overseas Finance Inventory Database—that track different sources and types of international development finance from China. All of these datasets have limited scope parameters^20–22^. Some exclusively track the overseas lending activities of China’s two policy banks: China Eximbank and China Development Bank. Others focus on a single sector or region. None provide a fully comprehensive picture of China’s overseas development program^2,23^. Nor do any of these datasets provide data on the spatio-temporal rollout of China’s overseas development projects that are sufficiently precise for causal inference.

To address this major evidentiary gap, the AidData research lab at William & Mary constructed a dataset—known as the Global Chinese Development Finance (GCDF) Dataset (Version 3.0)—that captures all sources and types of financial and in-kind transfers from government and state-owned institutions in China to 165 low- and middle-income countries^2,3^. It provides comprehensive coverage of all Chinese grant- and loan-financed projects (worth $1.34 trillion) across all regions and all sectors over 22 commitment years (2000–2021), as well as details on the timing of project implementation over a 24-year period (2000–2023). Projects are assigned 3-digit sector codes and categorized as Official Development Assistance (ODA) or Other Official Flows (OOF)—based on the measurement standards of the Development Assistance Committee of the Organisation for Economic Co-operation and Development (OECD-DAC)—to enable comparisons with other official sources of international development finance.

The dataset captures projects supported by 791 official sector donors and lenders based in China. At the project level, it also identifies the participation of 1,225 co-financing institutions—including Western commercial banks, multilateral development banks, and OECD-DAC development finance institutions that have chosen to collaborate or coordinate with official sector institutions in China. Additionally, the dataset identifies 5,037 receiving institutions, categorizing each one by type (government agency, state-owned bank, state-owned company, special purpose vehicle/joint venture, intergovernmental organization, private sector, etc.) and country of origin (recipient country, China, or a third country).

Another important feature of the dataset is its temporal granularity. It identifies precise, calendar day-level commencement dates for 11,286 projects and calendar day-level completion dates for 11,542 projects. These data are crucial for studies that seek to achieve causal identification because they allow for precise measurement of the timing of “treatment” exposure. For example, Wellner *et al*. (forthcoming)^10^ exploit the staggered rollout of the Gallup World Poll (GWP) and the availability of precise interview dates by comparing respondents who were interviewed in the month before and the month after the occurrence of a particular type of Chinese project “event.” They estimate the public opinion impacts of several different types of Chinese project “events,” including commencement dates and completion dates. They analyze these data with an event-study model that includes high-dimensional fixed effects. By using fixed effects at the country-level and province-year level and including a battery of variables that control for individual, geographical area, and survey characteristics, the timing of the GWP interviews can be considered as-if random, thereby providing a plausibly exogenous source of variation necessary for causal inference.

In pursuit of inferential leverage, a growing number of studies exploit spatio-temporal variation in treatment exposure to estimate the causal effects of Chinese grant- and loan-financed projects on social, economic, governance, and environmental outcomes at subnational scales. With earlier versions of the GCDF dataset that include substantially fewer projects with precise geocodes and commencement and completion dates, these studies seek to measure the localized effects of China’s overseas development projects on employment^24^, firm sales growth^25^, agricultural productivity^26,27^, household welfare^4^, economic growth and development^2,5,6,8^, nutrition^28^, infant mortality,^29^, environmental degradation^18,30^, political capture^15,31^, corruption^13,32^, acceptance of authoritarian norms^33^, acceptance of gender equality norms^34^, ethnic identification^35^, labor union participation^14^, satisfaction with public services^10^, popular support for China^10,36–39^, popular support for host governments^10,40^, tax morale and compliance^41,42^, media sentiment^43^, civic engagement^44^, public protest^17^, social stability^33^, and violent conflict^33^.

With case matching, difference-in-differences, fixed effects, and regression discontinuity techniques, most of these studies exploit variation in the timing of treatment exposure across geographic space. However, given that most of the “treatment” data were generated with non-trivial levels of spatial and temporal measurement imprecision, the researchers who conducted these studies generally relied on annual measures of treatment exposure and outcomes at the ADM1- or ADM2-level. For example, in earlier versions of the GCDF dataset, all of the provinces or districts intersecting with the route of a road may have been identified, but not the precise route between road’s start point and end point.

These sources of measurement imprecision are potentially consequential in that estimates of causal impact can be biased toward zero when treatment exposure is measured with a significant amount of error^30,45^. They also make it more difficult to identify heterogeneous treatment effects over space and time. A new generation of studies demonstrate that spatially and temporally heterogeneous treatment effects are easier to identify with calendar day-level or month-level measures of treatment exposure and/or measures of treatment exposure at small geographical scales (e.g., 1 km × 1 km grid cells)0^10,46,47^.

The publication of the 3.0 version of the GCDF dataset (hereafter referred to as GCDF v3) creates opportunities to address new questions and revisit old questions about the intended and unintended impacts of China’s grant- and loan-financed projects in the developing world. Yet a key limitation of the dataset is the absence of information about the precise geographical locations where projects take place. In the remainder of this study, we document our efforts to build a spatially explicit version of the dataset. The Geospatial Global Chinese Development Finance Dataset, Version 3.0 (hereafter referred to as Geo-GCDF v3) provides spatial information on 9,405 projects which have physical footprints or involve specific locations by extracting point, polygon, and line vector data from OpenStreetMap. 6,882 (or 73%) of these projects are precisely georeferenced (i.e., the physical boundaries of project sites are identified) or approximately georeferenced (i.e., within a 5 kilometer radius of the actual project site), while the remaining 2,523 (or 27%) are measured at an administrative unit level.

## Methods

In this section, we provide an overview of the data collection and processing steps used to produce the Geo-GCDF v3^48^. The methodology consists of two primary components: (1) data collection, which consists of identifying and recording—and editing, as needed—OpenStreetMap (OSM) elements associated with projects as well as descriptive locational information; and (2) data processing, which involves converting the raw OSM elements from their URLs and the descriptive locational information into standardized geospatial features.

### Data collection

The data collection process begins with the final release of the GCDF v3. The associated methodology document details the extensive set of validation and quality assurance processes that underpin the GCDF v3^3^. The initial step of the data collection process is identifying the subset of Chinese grant- and loan-financed projects that (a) support the construction, rehabilitation, upgrading, maintenance, expansion, or preservation of physical assets with identifiable geographical features, and/or (b) support activities which take place at specific locations with identifiable geographical features. The purpose of this step is to identify the ultimate geographical destinations of Chinese aid and credit. Examples of (a) include roads, railways, airports, seaports, power plants, electricity transmission lines, industrial parks, schools, hospitals, stadiums, and museums. Examples of (b) include medical teams stationed at a given hospital or equipment given to park rangers to patrol a demarcated protected area. Projects with no geospatial information available through project documentation or other sources—or projects without specific locational destinations—are not processed in the geospatial data collection process. To compile project locations, we leverage geospatial features defined by OpenStreetMap (OSM), which is a free, editable geographic database of the world that is built by volunteers and released with an open-content license (see: https://www.openstreetmap.org/copyright). In addition to utilizing existing features from the extensive data available in OSM, we contribute updates or additions for features that reflect project activities when it is necessary and feasible to do so.

To identify features within OSM that are associated with projects and features that need to be edited/added, we utilize the information in the “location narrative” field of the GCDF v3, the underlying sources and documentation that underpin the corresponding project records, and related sources to conduct targeted searches. The GCDF v3 was assembled with the 3.0 version of the Tracking Underreported Financial Flows (TUFF) methodology^3^. The TUFF methodology was developed through multiple rounds of scientific peer review over a 10-year period in collaboration with an international network of researchers from Harvard University, Heidelberg University, the University of Göttingen, the University of Cape Town, Brigham Young University, Georgetown University, the Kiel Institute for the World Economy, and William & Mary^2,7,15,19,49–51^. It provides a systematic, transparent, and replicable set of procedures that standardize and synthesize large volumes of unstructured information from four main sources: (1) data and documentation from Chinese ministries, embassies, and economic and commercial counselor offices; (2) the aid and debt information management systems of finance and planning ministries in counterpart countries; (3) case study and field research undertaken by scholars and NGOs; and (4) English, Chinese and local-language news reports. In total, the GCDF v3 is based on 147,703 sources in more than a dozen languages.

**Fig. 1:**
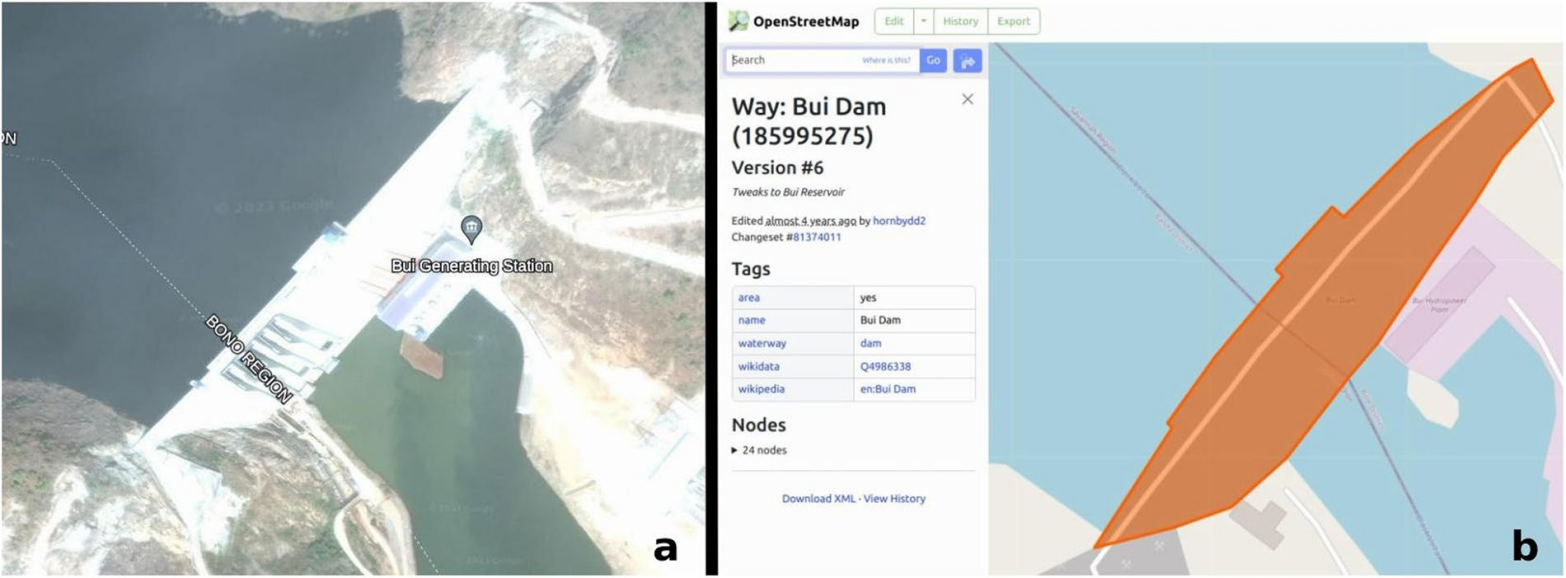
Feature identification example based on the Bui Dam in Ghana. See https://china.aiddata.org/projects/30801/ for more information about this project. (**a**) Imagery of dam (Source: Google Earth and Maxar; see: http://bit.ly/google_earth_bui_dam. (**b**) OpenStreetMap feature for dam (see https://www.osm.org/way/185995275).

In order to illustrate how features within OSM are associated with projects from the GCDF v3 consider a project description of a hydroelectric power station—such as the China Eximbank-financed Bui Dam in Ghana, shown in Fig. 1—built in the eastern section of a city along a river. The information provided in the “location narrative” field of the dataset is first cross-referenced with satellite imagery of the area as well as site photos (attached to the dataset record) to determine the project’s exact location (see Technical Validation section for more on the use of satellite imagery). We then search OSM for existing features associated with the hydroelectric power station, editing or adding new features if necessary. Finally, we record the corresponding features from OSM by saving the OSM URL, which contains the OSM element type (described further in Data Processing section) that is used to represent the feature and OSM ID.

Due to sometimes limited information available from project records, it is not always possible to identify a precise feature. However, an approximate location, or the boundary of an administrative zone may be available to fill the informational gap. Our data collection goal is to identify features at the most precise level, and then follow a hierarchy of order to fill in less precise location information if the most precise feature is not available. The system used in this process is defined as the level of precision (Table 1). After OSM URLs have been recorded, we refer to the level of precision system to label each project accordingly.

**Table 1:** Overview of levels of precision.

Level of Precision	Definition	Example
Precise	The precise boundary or footprint of a project’s activities are available.	The footprint of an airport or path of a highway.
Approximate	The precise location could not be identified, but an area or landmark used to reference the project and within 5 km of actual project location was found.	A power station is not identifiable with existing information but a nearby substation is identifiable in a 5 km radius.
Administrative Zone	No precise or approximate locations could be identified. The most precise administrative zone that can be found containing the project will be used instead, and the administrative level recorded. E.g., ADM4. See the *Usage Notes* section for more details on how administrative levels are defined in OSM.	Neither the power station nor any nearby features could be found, but it was known which district (ADM6) the power station was constructed in. The boundary feature associated with the district is then used to represent the project.

Once OSM URLs and the level of precision for a project are identified, the information is reviewed and quality-assured by a second party to verify that the feature accurately reflects the final destination of the source of Chinese aid/credit and the OSM feature collected is at the most precise level available. Both OSM features and the level of precision system are cross-checked with project records during the review.

After these initial collection and review steps are completed, we run automated checks to identify human errors and invalid OSM URLs. Potential errors include invalid OSM URLs (typically either the result of a removed OSM element due to further edits on the OSM data platform from other collaborators, or copying an incomplete URL from a browser), missing or mismatched combinations of OSM URLs and levels of precision (e.g., 3 URLs are collected for a project but only 2 levels of precision are recorded), and other typos or errors. Projects with issues during this stage are flagged for a second review and troubleshooting. Once all of the identified issues are addressed and all projects pass the validation checks, the collected data is automatically formatted and can continue to the processing stage.

### Data processing

The goal of the data processing stage is to extract the OSM features from the URLs recorded, and convert them into a standardized geospatial format compatible with most GIS and geospatial analysis tools and software. The first step in the data processing workflow is retrieving the raw OSM elements using a combination of web scraping and the Overpass API. (Overpass provides an API for accessing OSM features that can be used with Python and other programming language; see the Overpass API wiki page and the Overpass API Python wrapper GitHub repository).

Additional processing of OSM features is necessary as OSM uses a unique data schema. Features in OSM can be represented by 3 types of elements: nodes, ways, and relations. We treat driving/routing directions as a separate fourth type that are utilized to represent linear features along roads or other routes where it would be less practical or precise to represent them using OSM element. Roads and other linear features associated with projects can often be (1) represented by many constituent OSM elements, or (2) represented by a single element that is larger than desired for the project. Rather than recording dozens of individual elements or recording a larger and less accurate element, driving directions that traverse the complete and precise route can be used.

Nodes define points, while ways define linear features (i.e., a line or open way) or area boundaries (i.e., a polygon or closed way). Relations provide a more complex description of how other elements can be combined together; they can include arbitrary combinations of nodes, ways, and other relations as members. The OSM elements are an effective way to represent geospatial features in a massive and flexible dataset intended to represent any type of feature users may be interested in recording. Individual elements—or even existing relations built upon multiple other elements—implemented by one user can be leveraged by another to form more complex representations. A case in point is how ways are used to represent the borders of a country: the same ways representing parts of the country border can be combined with additional ways in order to represent the borders of states within the country. However, the frequent complexity of relations combining many constituent elements can make OSM features representations difficult to translate into traditional representations of geospatial features.

Given the nature of relations and the fact that an individual project may consist of multiple OSM features, it is possible that multiple geospatial feature types (points, lines, polygons, and multi-feature versions of each) are used to represent a single project. This presents a challenge when attempting to produce a uniform data model for the Geo-GCDF v3^48^. We attempt to convert the raw OSM elements retrieved into traditional vector geospatial features primarily by using the osm2geojsonpackage in Python. Features defined using driving/routing directions use a different method for extracting the feature information, which involve web scraping the map produced at associated OSM link (and future versions of the Geo-GCDF v3 will generate similar feature information by using the OSRM routing engine).

This conversion step is the most common source of error during the data processing stage. Errors usually result from one of two problems. The first type of error is when the associated OSM element has been changed or deleted since it was recorded. The second type of error is associated with the potential ambiguity of converting OSM elements to traditional vector geospatial features, as they can rely on information from *tags* or *roles* associated with the OSM element to inform how the conversion takes place. Due to the flexible schema of OSM, tags and roles are not strictly enforced and may be missing or incorrectly used. A common example of this is when using a series of ways to define a relation representing a polygon feature. If the ways representing the exterior edge of the polygon are not defined with roles of *outer*, the conversion in osm2geojson may produce a LineString feature rather than a Polygon. Uses of tags such as *multipolygon* also facilitate determining the intended representation of features in OSM.

The errors identified at this step are sent back for review so that an appropriate OSM element can be found if the original was changed/deleted, or the underlying OSM element is edited to correct any issues identified. Once all issues with OSM elements have been addressed, the resulting geospatial features are cached as individual GeoJSON files. Caching at this stage prevents unnecessarily querying the Overpass API or performing redundant and potentially time-consuming data processing. In addition, since OpenStreetMap changes frequently as users submit new changes, rebuilding this dataset by querying for the same feature IDs may result in missing features or different geometries. Caching effectively produces a snapshot of relevant OSM features at the time of data processing, and avoids continuously modifying the dataset to adapt to changes. Cached features, which are made publicly available along with the dataset through the GitHub repository, are also critical to supporting replication.

The converted OSM elements are now represented as traditional vector geospatial feature types—Points, LineStrings, Polygons, and MultiPolygons. ESRI’s ArcGIS Resource Center provides additional details on these types of geospatial features. To standardize the features into a single feature type for ease of use, all point and line features are buffered slightly (approximately one meter) to transform them into polygons. By ensuring all features are polygons, we are able to account for multiple features associated with a single project by combining all polygons into a single multi-polygon feature. In order to avoid producing invalid geometries, such as those that have self-intersections, all polygons associated with a project are dissolved prior to generating the final MultiPolygon. The raw vector geospatial features (i.e., LineStrings for roads) are saved and also made available to users (see the section on Raw OSM Features in the Usage Notes/Supporting Data).

The resulting MultiPolygon features are then joined with a limited subset of data fields from the GCDF v3 and saved as individual GeoJSON files as well as combined into a single GeoPackage file to support use in a range of applications.

## Data Records

These data records describe the contents of the Geo-GCDF v3^48^, including file formats and specific fields available within the dataset. The dataset at the time of publication is archived and available via Figshare. In addition, the dataset—along with future updates and all historical versions, example code, documentation, and other features—is available through a repository hosted on GitHub (https://github.com/aiddata/gcdf-geospatial-data).

### Repository

The Geo-GCDF v3^48^ is made available via Figshare as well as a public GitHub repository. On GitHub, the Geo-GCDF v3 will be made available through the Releases page of the GitHub repository, which is used to permanently archive versions of the dataset. The data in the core repository may be continually updated as improvements are made, and future versions of the dataset may be added to the *Releases* page as well.

The specific version of the Geo-GCDF Dataset follows semantic versioning standards with the major and minor version mirroring the release of the GCDF v3 (see: https://semver.org/). The patch version is unique to changes within the Geo-GCDF that may reflect adjustments to code and processing of the geospatial features, but not changes in the underlying project data (from GCDF v3). Each release version using semantic version will be of the format *Release v3.0.x*.

In addition to official releases of the dataset, the GitHub repository contains the code and data necessary to replicate creation of the final product, as well as examples and documentation for running the code and working with the dataset. Details on replication steps, guidelines for modifying the code for new input data, and more can be found in the README file within the root directory of the repository.

### Data files

Geospatial features and information associated with each project are made available in two formats. The first is as a GeoPackage file, which combines all features in a single layer (see: https://www.geopackage.org/). The second is a collection of individual GeoJSON files (see: https://geojson.org/). The data records, including the geospatial feature definitions and additional attribute fields, are identical in both formats. The compressed GeoPackage file (all_combined_global.gpkg.zip) is available through the Figshare repository as well as through the GitHub repository, where it is attached directly to a specific GitHub release and available as a direct download from a given release’s page.

The GeoPackage file containing the complete collection of features is a single, large file (approximately 500MB compressed in a ZIP file and 730MB uncompressed) that provides fast access to all of the data. When exploring the data, performing custom queries, or conducting large-n analysis, using the GeoPackage file will likely be the preferred approach. The individual GeoJSON files are, on average, much smaller files and well-suited to applications that only require a small subset of the data. However, all of the individual GeoJSON files (nearly 2GB) are noticeably slower when they are simultaneously read into GIS software or accessed with code.

The GeoJSON files also have the benefit of being individually accessible with direct GitHub links, which can be used to visualize the features on GitHub in a web browser (for an example, see https://gist.github.com/sgoodm/dafd02fb4d610b334fb75d9a319d898b). The direct links can be generated based purely on the *id* field associated with each data record, allowing flexible access for specific applications (e.g., generating links within a custom website application to download and access specific GeoJSONs on demand). The link format also allows users of the GCDF v3 to easily find associated GeoJSON files. The GeoJSON files are also available through the Figshare repository as part of the ZIP file containing the full contents of the GitHub repository (gcdf-geospatial-data-3.0.0.zip).

### Core data fields

The core data fields are produced to describe the location of associated projects from the GCDF v3. Records in the Geo-GCDF v3^48^ can be associated with records in the GCDF v3 using the *id* and *AidData Record ID* fields, respectively.

#### Data Field: geometry

Each data record is defined by a single MultiPolygon feature that indicates the geospatial location of the associated project’s activities (see https://shapely.readthedocs.io/en/stable/reference/shapely.MultiPolygon.html for MultiPolygon reference).

#### Data Field: id

The *id* field corresponds to the GCDF v3 *AidData Record ID*. This field provides the unique identification number that AidData has assigned to each project/activity record in the dataset.

#### Data Field: feature_count

An integer value that indicates the number of constituent OSM elements which were used to build the final geospatial feature (see: geometry)

#### Data Field: osm_links

A comma-separated list of the links to each OSM element which was used to build the final geospatial feature. An example OSM link: https://www.openstreetmap.org/node/299617915)

#### Data Field: osm_precision_list

A comma-separated list of the precision associated with each OSM element used to build the final geospatial feature. The order of the items in the *osm_precision_list* correspond to the order of the items in the *osm_links* field. Example precision values: precise, approximate, adm1, adm2. Over 6,200 projects ( > 66%) are represented by precise features and reflect more than 75% of the total commitment value of projects in the Geo-GCDF v3. Approximately 600 projects (which reflect an additional 12% of the total commitment values) are represented by approximate features, within 5 km of the true location. The remaining approximately 2,500 projects ( < 27%) are represented by varying administrative level boundaries and detail only around 10% of project commitment values covered by the Geo-GCDF v3. The full breakdown of project counts and commitment values by feature precision are detailed in Table 2.

**Table 2:** Project count and commitment value by precision level.

Precision	Count	Count %	Value $	Value %
precise	6266	66.62	637.73	76.75
approximate	616	6.55	102.64	12.35
adm11	3	0.03	0.00	0.00
adm10	6	0.06	0.01	0.00
adm9	49	0.52	2.20	0.26
adm8	597	6.35	18.96	2.28
adm7	99	1.05	2.41	0.29
adm6	516	5.49	28.30	3.41
adm5	176	1.87	3.07	0.37
adm4	1032	10.97	34.88	4.20
adm3	45	0.48	0.70	0.08

Value in billions of 2021 USD.

### Ancillary data fields

Thirteen ancillary data fields are pulled directly from the GCDF v3 to provide additional utility to users of the Geo-GCDF v3^2,3,48^. Joining additional fields from the GCDF v3 is discussed in the Usage Notes section.

#### Data Field: recipient

This field captures the country from which the entity receiving the official financial or in-kind transfer is located. If entities from multiple recipient countries are involved, this field records the geographical region to which the recipient countries belong. The Geo-GCDF v3 covers a total of 148 low and middle income countries, with the number of countries covered in a given year ranging from 58 (in 2000, the first year of data collected), up 126 in 2018. The full breakdown of country coverage by year is detailed in Table 6.

#### Data Field: recipient.ISO-3

This field captures the three-letter code for the country identified in the “Recipient” field, according to the standards set by the International Organization for Standardization (ISO). In cases where the “Recipient” field records the geographical region from which multiple recipient countries belong (such as “Africa, Regional”), the ‘Recipient ISO Alpha-3 Country Code’ field is left blank.

#### Data field: title

This field briefly describes the name or nature of the project/activity. The identification numbers of other transactions that are linked to the project/activity are also recorded in this field.

#### Data Field: commitment.year

This field captures the year in which an official financial commitment (or official commitment to provide in-kind support) was codified through the signing of a formal agreement by an official donor/lender in China and one or more entities in a recipient country or a set of recipient countries. Whenever possible, this field is based on the precise calendar day when the official commitment was issued, which is captured in the “Commitment Date” field. In the event an official commitment was made for a project/activity that entered implementation, but the official commitment year is not identifiable, AidData records the first year of project/activity implementation as a proxy for the official commitment year. In the event an official commitment was made for a project/activity that has not yet reached implementation, and the official commitment year is not identifiable, AidData records the year in which the underlying commercial contract (supported by the official commitment) was issued. If this information is unavailable, AidData records the first year in which an informal pledge was made as a proxy for the official commitment year. For projects/activities with a status designation of “Pipeline: Pledge” (i.e. cases in which an official commitment was not made), AidData records the year in which the informal pledge was made.

The breakdown of project counts and commitment values by year, including percentages as breakdown of totals, are detailed in Table 3. It enables calculations of average project sizes by dollar value over time, and year-on-year changes in their size and scale.

**Table 3:** Project count and commitment value by year.

Year	Count	Count %	Value $	Value %
2000	115	1.22	4.70	0.57
2001	121	1.29	4.08	0.49
2002	138	1.47	4.88	0.59
2003	164	1.74	8.32	1.00
2004	175	1.86	6.85	0.82
2005	263	2.80	20.01	2.41
2006	317	3.37	23.69	2.85
2007	338	3.59	19.29	2.32
2008	360	3.83	28.57	3.44
2009	420	4.47	99.90	12.02
2010	451	4.80	52.30	6.29
2011	475	5.05	37.33	4.49
2012	430	4.57	47.39	5.70
2013	470	5.00	90.81	10.93
2014	489	5.20	57.88	6.97
2015	577	6.14	49.59	5.97
2016	692	7.36	90.10	10.84
2017	760	8.08	64.32	7.74
2018	916	9.74	40.81	4.91
2019	767	8.16	41.20	4.96
2020	441	4.69	10.64	1.28
2021	526	5.59	28.24	3.40

Value in billions of 2021 USD.

#### Data Field: implementation.start.year

This field captures the year in which a project/activity supported by an official financial (or in-kind) commitment from China began implementation. Whenever possible, this field is based on the precise calendar day when project/activity implementation began, which is captured in the “Actual Implementation Start Date” field. For projects/activities that involve the construction of buildings or infrastructure, the “Implementation Start Year” field seeks to capture the first year of construction. In cases when the first year of construction is unavailable but a proxy for the first year of construction (e.g., the year in which a formal groundbreaking ceremony took place, a project/activity commencement order was issued to the contractor responsible for implementation, or a project/activity implementation agreement was signed) can be identified, AidData records the proxy for the first year of construction. For projects/activities that do not involve construction but involve the provision of personnel, training, analytical/advisory support, equipment, supplies, or commodities, the “Implementation Start Year” field captures the first year in which some type of support was delivered to an entity in the recipient country. For projects/activities that only involve financial transactions (e.g., cash donations, loans issued to shore up a country’s foreign exchange reserves, forgiveness or rescheduling of outstanding debts), the “Implementation Start Year” field captures the year in which the first disbursement was made (or the year in which new terms and conditions went into effect for a previously signed loan agreement).

#### Data Field: completion.year

This field captures the year in which a project/activity supported by an official financial (or in-kind) commitment from China was completed. Whenever possible, this field is based on the precise calendar day when a project/activity was completed, which is captured in the “Actual Completion Date” field. For projects/activities that involve the construction of buildings or infrastructure, the “Completion Year” field seeks to capture the last year of construction. In cases when the last year of construction is unavailable but a proxy for the last year of construction (e.g., a road or railway is opened for use, a power plant reaches its commercial operation date and begins selling electricity to customers) can be identified, AidData records the proxy for the last year of construction. For projects/activities that do not involve construction but involve the provision of personnel, training, analytical/advisory support, equipment, supplies, or commodities, the ‘Completion Year’ field captures the last year in which some type of support was delivered to an entity (or set of entities) in the recipient country. For projects/activities that only involve financial transactions (cash donations, loans issued to shore up foreign exchange reserves, forgiveness or rescheduling of outstanding debts), the ‘Completion Year’ field captures the year in which the last disbursement was made (or the year in which new terms and conditions went into effect for a previously signed loan agreement).

#### Data Field: commitment.date.(MM/DD/YYYY)

This field seeks to capture the day on which an official financial commitment (or official commitment to provide in-kind support) was codified through the signing of a formal agreement by an official donor/lender in China and one or more entities in a recipient country or a set of recipient countries. Whenever possible, this field is based on the precise calendar day on which the official commitment was made. However, in cases when AidData is only able to identify the month and year in which the formal agreement was signed (e.g. May 2018), the “Commitment Date” field is set to the first day of the month (01/01/2018). In cases when AidData is only able to identify the year in which the formal agreement was signed, the “Commitment Date” field is set to the first day of the first month (e.g. 01/01/2018). In the event an official commitment was made for a project/activity that entered implementation, but the official commitment year is not identifiable, AidData records the first year of project/activity implementation as a proxy for the official commitment year. In the event an official commitment was made for a project/activity that has not yet reached implementation, and the official commitment year is not identifiable, AidData records the year in which the underlying commercial contract (supported by the official commitment) was issued. If this information is unavailable, AidData records the first year in which an informal pledge was made as a proxy for the official commitment year. For projects with a status designation of Pipeline Pledge (i.e. cases in which an official commitment was not made), AidData records the date on which the informal pledge was made.

#### Data Field: actual.implementation.start.date.(MM/DD/YYYY)

This field seeks to capture the day on which a project/activity supported by an official financial (or in-kind) commitment from China began implementation. Whenever possible, this field is based on the precise calendar day when project/activity implementation began. However, in cases when AidData is only able to identify the month and year in which project/activity implementation began (e.g., May 2018), the “Actual Implementation Start Date” field is set to the first day of the month (e.g. 05/01/2018). For projects/activities that involve the construction of buildings or infrastructure, the “Actual Implementation Start Date” field seeks to capture the first day of construction. In cases when the first day of construction is unavailable but a proxy for the first day of construction (e.g., the date on which a formal groundbreaking ceremony took place, a project/activity commencement order was issued to the contractor responsible for implementation, or a project/activity implementation agreement was signed) can be identified, AidData records the proxy for the first date of construction. For projects/activities that do not involve construction but involve the provision of personnel, training, analytical/advisory support, equipment, supplies, or commodities, the “Actual Implementation Start Date” field captures the first day in which some type of support was delivered to an entity (or set of entities) in the recipient country. For projects/activities that only involve financial transactions (cash donations, loans issued to shore up a country’s foreign exchange reserves, forgiveness or rescheduling of outstanding debts), the “Actual Implementation Start Date” field captures the day on which the first disbursement was made (or the day on which new terms and conditions went into effect for a previously signed loan agreement).

#### Data Field: actual.completion.date.(MM/DD/YYYY)

This field seeks to capture the day on which a project/activity supported by an official financial (or in-kind) commitment from China was completed. Whenever possible, this field is based on the precise calendar day when a project/activity was completed. However, in cases when AidData is only able to identify the month and year in which a project/activity was completed (e.g., May 2018), the “Actual Completion Date” field is set to the first day of the month (e.g., 05/01/2018). For projects/activities that involve the construction of buildings or infrastructure, the “Actual Completion Date” field seeks to capture the last day of construction. In cases when the last day of construction is unavailable but a proxy for the last day of construction (e.g., a road or railway is opened for use, a power plant reaches its commercial operation date and begins selling electricity to customers) is available, AidData records the proxy for the last day of construction. For projects/activities that do not involve construction but involve the provision of personnel, training, analytical/advisory support, equipment, supplies, or commodities, the “Actual Completion Date” field captures the last day on which some type of support was delivered to an entity (or set of entities) in the recipient country. For projects/activities that only involve financial transactions (cash donations, loans issued to shore up foreign exchange reserves, forgiveness or rescheduling of outstanding debts), the “Actual Completion Date” field captures the day on which the last disbursement was made (or the day on which new terms and conditions went into effect for a previously signed loan agreement).

#### Data field: amount.(Constant.USD.2021)

This field captures the monetary value of the official commitment (or pledge) issued by the funding agency in constant 2021 U.S. dollars. To calculate this value, AidData first converts the financial commitment (or pledge) amount in its original currency of denomination to nominal U.S. dollars at the average exchange rate in effect during the commitment (or pledge) year, and then converts this amount to constant 2021 U.S. dollars using the OECD’s deflation methodology to adjust for inflation and ensure comparability over time and space.

#### Data field: status

This field identifies the latest status of a project/activity. Each project/activity is assigned to one of six categories: “Pipeline: Pledge,” “Pipeline: Commitment,” “Implementation,” “Completed,” “Suspended,” or “Cancelled.” A project/activity assigned to the “Pipeline: Pledge” category is one that an official sector institution in China indicated it was interested in supporting (or willing to consider supporting) but did not result in an official commitment. Projects/activities assigned to this category include those that are identified in letters of intent, term sheets, memoranda of understanding, and non-binding announcements. All projects/activities given a status designation of “Pipeline: Commitment,” “Implementation,” “Completed,” “Suspended,” or “Cancelled” reached the official commitment stage (i.e., a binding, written agreement that governs the provision of financial or in-kind support for a specific purpose was signed by an official sector donor or lender in China and an entity in a recipient country). A project/activity assigned to the “Pipeline: Commitment” category is one that is backed by an official commitment but has not yet entered implementation. A project/activity assigned to the “Implementation” category is one that is backed by an official commitment and has begun implementation with financial or in-kind support from the source of the commitment. A project/activity assigned to the “Completion” category is one that is backed by an official commitment and that reached completion with financial or in-kind support from the sources of the commitment. Projects/activities assigned to the “Suspended” and “Cancelled” categories are those that were backed by an official commitment but subsequently suspended or cancelled. The coding of the “Status” field in the dataset is based on sources that were available as recently as August 2023.

#### Data field: sector.name

This field provides a sector name based upon the primary sectoral focus of the project/activity. It is based upon the OECD’s sector categorization scheme. There are 24 OECD sectors (OECD sector codes in parentheses): education (110), health (120), population policies/programs and reproductive health (130), water supply and sanitation (140), government and civil society (150), other social infrastructure and services (160), transport and storage (210), communications (220), energy (230), banking and financial services (240), business and other services (250), agriculture, forestry and fishing (310), industry, mining, and construction (320), trade policies and regulation (330), general environmental protection (410), other multisector (430), general budget support (510), developmental food aid/food security assistance (520), other commodity assistance (530), action relating to debt (600), emergency response (720), reconstruction relief and rehabilitation (730), disaster prevention and preparedness (740), and unallocated/unspecified (998).

Table 4 details the breakdown of project values and counts by year and sector, with an emphasis on the Industry, Energy, and Transport sectors.

**Table 4:** Commitment values by sector and year for major sectors.

Year	Industry	Energy	Transport	Comms	Other	Total
2000	1.72 (24)	1.22 (12)	0.04 (3)	0.09 (6)	1.62 (70)	4.69 (115)
2001	0.43 (10)	1.44 (11)	1.48 (16)	0.01 (6)	0.70 (78)	4.06 (121)
2002	0.32 (8)	1.29 (11)	2.43 (19)	0.16 (6)	0.67 (94)	4.87 (138)
2003	0.60 (11)	5.53 (16)	1.13 (15)	0.11 (8)	0.94 (114)	8.31 (164)
2004	0.13 (7)	2.05 (14)	3.94 (22)	0.03 (7)	0.71 (125)	6.86 (175)
2005	6.91 (9)	6.23 (18)	1.71 (12)	1.47 (16)	3.68 (208)	20.00 (263)
2006	10.76 (23)	4.60 (24)	3.27 (27)	1.14 (18)	3.91 (225)	23.68 (317)
2007	3.72 (13)	5.16 (26)	7.66 (44)	0.41 (15)	2.34 (240)	19.29 (338)
2008	15.04 (22)	5.49 (31)	2.87 (32)	1.50 (10)	3.66 (265)	28.56 (360)
2009	65.71 (36)	18.46 (40)	7.76 (50)	2.46 (18)	5.50 (276)	99.89 (420)
2010	14.81 (24)	18.15 (60)	6.28 (60)	1.68 (10)	11.38 (297)	52.30 (451)
2011	11.99 (32)	8.21 (53)	6.96 (58)	5.71 (24)	4.46 (308)	37.33 (475)
2012	15.09 (38)	7.45 (49)	9.66 (50)	1.91 (17)	13.27 (276)	47.38 (430)
2013	52.85 (32)	20.61 (78)	12.20 (59)	0.19 (7)	4.96 (294)	90.81 (470)
2014	16.88 (42)	19.80 (68)	13.32 (49)	0.87 (6)	7.00 (324)	57.87 (489)
2015	13.05 (45)	18.07 (66)	9.78 (68)	2.31 (18)	6.38 (380)	49.59 (577)
2016	32.52 (65)	32.63 (79)	14.58 (106)	0.47 (12)	9.90 (430)	90.10 (692)
2017	9.63 (59)	26.89 (93)	17.54 (75)	0.62 (18)	9.63 (515)	64.31 (760)
2018	12.03 (45)	10.50 (40)	12.57 (85)	1.13 (24)	4.56 (722)	40.79 (916)
2019	8.26 (35)	7.44 (45)	19.71 (62)	1.11 (15)	4.68 (610)	41.20 (767)
2020	1.62 (9)	5.81 (19)	1.29 (15)	0.15 (4)	1.76 (394)	10.63 (441)
2021	15.10 (39)	3.13 (30)	5.21 (30)	0.36 (8)	4.43 (419)	28.23 (526)

Values are in billions of 2021 USD with count of projects in parentheses. “Other” contains 17 additional sectors not listed.

#### Data field: infrastructure

This flag provides a marker of whether a project/activity is an infrastructure project. In the 3.0 version of the dataset, “infrastructure projects” generally include those that involve physical construction activities (e.g. roads, railways, pipelines, transmission lines, fiber optic networks). More specifically, “infrastructure projects” include those that involve (1) building a new physical structure, (2) rehabilitating or adding onto an existing physical structure, and/or (3) maintaining an existing physical structure. The 3.0 version of the dataset does not include projects/activities that involve the provision of cash, technical assistance, scholarships, equipment, or supplies in its definition of “infrastructure projects.” The field is set to “Yes” if a project/activity is classifiable as an infrastructure project.

## Technical Validation

### Data collection

The data collection stage incorporates two primary validation elements, referenced in the Methods section. First, all precise project locations are verified using satellite imagery (see Fig. 1). Less precise project locations also use imagery alongside other sources to identify the approximate location or relevant administrative zone. Imagery is used from a variety of sources including Google Maps, Bing Maps, Google Earth, and other publicly available imagery. The specific imagery used is typically dependent on the time frame imagery was collected in relation to when projects were implemented or completed. Given the nature of the dataset, particularly for projects containing precise features, end users can typically load features alongside their own imagery to validate and explore the associated area themselves.

Second, all data collected is reviewed by at least two individuals. Although this is not a double-blind review procedure, the use of satellite imagery to verify project locations results in far less uncertainty when compared to previous approaches to geocoding where locations were selected entirely based on text descriptions^52^.

#### Data processing

Validation during the data processing stage is integrated into the data processing workflow presented in the Methods section. As a result, projects that do not meet all criteria cannot proceed through the entire workflow. The exact validation checks are documented within the code used for data processing and include confirming that there are no duplicates in the dataset; OSM URLs are resolvable and contain valid OSM elements; OSM links are paired with valid precision levels; OSM elements produce valid, non-empty geospatial features; and all geospatial features can be converted to MultiPolygons.

## Usage Notes

The Geo-GCDF v3^48^ can be utilized with most software, tools, and approaches that support standard geospatial data formats. Desktop GIS software, such as the open source QGIS platform or ESRI’s ArcGIS Pro, support a broad range of mapping, analysis, and other applications. Web-based platforms, such as MapBox and ArcGIS Online, also provide a range of functionality, often more tailored to sharing and visualizing outputs. Many popular programming languages have libraries or packages available which support working with geospatial data. In particular, Python has a large community supporting packages for many different applications of geospatial data, ranging from visualization to machine learning. See geopandas and shapely for working with geospatial features, rasterio and python-rasterstats for incorporating raster data, Folium and Ipyleaflet for mapping, and torchgeo for deep learning. Many other common Python packages such as Numpy and SciKit-Learn can be useful for working with spatial data.

In addition, the individual GeoJSON files hosted in the Geo-GCDF v3 data repository on GitHub can leverage built in GitHub functionality for exploring geospatial data. When visiting the page for any GeoJSON file in the repository (e.g., https://github.com/aiddata/gcdf-geospatial-data/blob/main/latest/geojsons/20000.geojson), GitHub will automatically render a basic visualization of the feature contained in the GeoJSON. This functionality can be useful to users who want to explore a small number of projects’ geospatial features, and do not necessarily need to download GIS software, create an account for a web mapping platform, or write custom code.

To support usage of the Geo-GCDF v3, a series of examples consisting of code, documentation, and guides are provided within the data repository on GitHub (see the ‘‘/examples’’ subdirectory: https://github.com/aiddata/gcdf-geospatial-data/tree/main/examples). The examples include several introductory topics, such as a visual overview of the data and features; loading the features and performing basic spatial operation using Python; and building maps using the features in QGIS. More advanced examples using Python are also provided, which demonstrate how to rasterize features based on their associated project commitment values and how to evaluate the change in levels of nighttime light output (luminosity) around project sites over time.

### Joining and filtering data

The full set of (more than 120) fields from the GCDF v3^2,3^ can be joined with the Geo-GCDF v3^48^ using the *AidData Recipient ID* field from the former and the *id* field from the latter. The fields included in the Geo-GCDF v3 are either (a) produced during the geospatial data collection process (see Core Data Fields section), or (b) pre-joined from the GCDF v3 (see Ancillary Data Fields section) to facilitate spatio-temporal analysis (i.e., recipient country, commitment date, implementation start date, and completion date). A small number of ancillary fields from the GCDF v3 are also included to support basic data exploration (title, status, sector name, and infrastructure).

Prior to performing any analysis, it is essential to read the methodological documentation and user guidance for the GCDF v3 and/or Geo-GCDF v3. Subsets of data may not be appropriate for analysis at certain levels of aggregation. For example, depending on the nature of the spatial analysis being conducted, the use of only precisely identified locations may be required. Approximate or administrative level features may also need to be excluded by using the “osm_precision_list” field to remove any projects containing the “adm” or “approximate” values in the list.

Users should also keep in mind that the GCDF v3 seeks to capture all Chinese grant- and loan-financed projects and activities that meet the OECD-DAC’s measurement criteria for Official Development Assistance (ODA) or Other Official Flows (OOF). Yet not all Chinese ODA- and OOF-financed projects and activities are appropriate for geospatial analysis (e.g., borrowings via currency swap arrangements, balance of payments support, debt forgiveness, commodity donations). Consequently, the Geo-GCDF v3 only provides geospatial features for the subset of projects in the GCDF v3 that have physical footprints (e.g., roads, railways, transmission lines) or involve activities at specific locations (e.g., medical teams stationed at a given hospital, equipment given to park rangers to patrol a well-demarcated protected area).

Finally, with respect to decision-making and policy-related applications of the Geo-GCDF v3 that are specific to individual Chinese ODA- or OOF-financed projects, we strongly encourage visual verification (with up-to-date satellite imagery) by users to ensure that the geospatial features contained in the Geo-GCDF v3 are accurate and up-to-date. Visual verification is especially important for those projects and activities that had not yet reached completion by 2023—the last year in which OSM features were retrieved for the construction of the Geo-GCDF v3.

### Interpreting administrative boundary features from OSM

The definitions of administrative divisions or levels are highly variable from country to country, with differing naming conventions, number of levels, and roles of the associated level (e.g., local laws or governance). For practical usage, administrative levels are often simplified to “first administrative level” (ADM1), “second administrative level” (ADM2), and “third administrative level” (ADM3), with subsequently finer levels utilized as needed. Examples of this include major products that define the geospatial boundaries of administrative divisions, such as geoBoundaries^53^ or GADM.

As a community-driven, volunteer geographic information (VGI) platform, OSM is highly dependent on local efforts to accurately map features. Country- or region-specific groups can provide informal governance and help organize efforts in their area, establish conventions for user contributions, and generally ensure the data in OSM reflects the reality on the ground for that area. While the local focus of OSM groups supports effective and accurate contributions, it can also result in different conventions between countries or regions. Variations in conventions are particularly notable when dealing with administrative levels. It is uncommon that any two countries have the exact same conventions for incorporating administrative features within OSM (see: https://wiki.openstreetmap.org/wiki/Tag:boundary%3Dadministrative).

With a notable subset of projects represented as administrative zones (see Table 1), OSM’s representation of these boundaries is of particular interest. OSM may contain as many as eleven distinct levels for some countries, in contrast to the two to five levels seen in datasets such as geoBoundaries or GADM. The additional boundaries follow unique definitions intended to support nuances of local administrative division. Most commonly, OSM ADM4 corresponds to the traditional ADM1, while OSM ADM6 corresponds to the traditional ADM2 and OSM ADM8 corresponds to the traditional ADM3. However, there are many exceptions to even these common administrative level definitions in OSM.

For example, in Ghana: *Regions* are defined as ADM4 in OSM and ADM1 in geoBoundaries; *Districts* are defined as ADM6 in OSM and ADM2 in geoBoundaries; *Towns/Villages* are defined as ADM8 in OSM and not included in geoBoundaries. While in Malawi: *Regions* are defined as ADM3 in OSM and ADM1 in geoBoundaries; *Districts* are defined as ADM4 in OSM and ADM2 in geoBoundaries; *Traditional Authorities/Urban Administrative Wards* are defined as ADM6 in OSM and ADM3 in geoBoundaries; *Villages* are defined as ADM10 in OSM and not included in geoBoundaries. The variability and potential complexity of utilizing administrative level definitions in OSM are increased given that there is no strict enforcement of the community definitions created.

Given these considerations, we suggest not treating the administrative levels and administrative features themselves as accurate administrative boundary definitions, but as the most precise feature that we could identify in OSM (for which we were certain the project’s activities were confined to). Users who intend to utilize features representative of the exact location of a project’s activities should filter the dataset to use only “precise” features. Users interested in only administrative level data, based on standardized administrative boundaries, can leverage an additional data product we produce and describe later in the Supporting Data section.

### Citing the dataset

The Geo-GCDF v3^48^ is extracted from OpenStreetMap, which is licensed under the Open Data Commons Open Database License (ODbL). Any use of this dataset must include appropriate attribution to OpenStreetMap; instructions for doing so can be found here.

Uses of the geospatial features from the Geo-GCDF v3 (see *Core Data Fields* in Data Records section) should cite this publication. Ancillary Data Fields produced by AidData and included in the Geo-GCDF v3 are licensed under the Open Data Commons Attribution License (ODC-By). Use of this data must include appropriate attribution of the GCDF v3^2,3^.

### Supporting data

#### Raw OSM features

Standardizing the geospatial features in this dataset as MultiPolygons facilitates general uses. However, specific use cases may necessitate, or be made significantly easier, by using the original unmodified features from OSM. Examples of such applications include network analysis of linear features such as roads. To support these applications, we separately provide the original OpenStreetMap features. Each geometry type (Point, LineString, Polygon, MultiPolygon) is saved as a separate GeoPackage file. The set of GeoPackage files are included in a ZIP file (OSM_grouped.zip) available in the Figshare repository, as well as attached to the dataset release available through the GitHub repository. The breakdown of feature by feature type is detailed in Table 5.

**Table 5:** Project count and commitment value by feature types.

Feature Type	Count	Count %	Value $	Value %
LineString	812	8.63	180.61	21.74
MultiPolygon	481	5.11	52.65	6.34
Point	1536	16.33	53.90	6.49
Polygon	6576	69.92	543.75	65.44
Total	9405	100.00	830.90	100.00

Value in billions of 2021 USD.

#### Administrative level data

A set of data products were also produced to support users who do not need to leverage full precision of the geospatial features in the Geo-GCDF v3^48^. These products include the list of administrative units in which projects took place. The data is produced by identifying intersecting ADM1 or ADM2 units from geoBoundaries v6 CGAZ layers^53^. For each Geo-GCDF v3 project, all intersecting ADM units are recorded and the proportion of the project’s geospatial feature which intersects with the ADM unit. The total amount of ADM1 and ADM2 units covered by the dataset are detailed in Table 6.

**Table 6:** Number of countries, ADM1 units, and ADM2 units covered in each year.

Year	Country	ADM1	ADM2
2000	58	238	939
2001	58	177	482
2002	71	229	684
2003	74	260	794
2004	82	283	1482
2005	81	500	2810
2006	95	400	1413
2007	99	423	1562
2008	99	471	1678
2009	109	487	1510
2010	110	552	2100
2011	106	558	1832
2012	110	461	1538
2013	110	478	1372
2014	117	629	3371
2015	118	604	2612
2016	123	574	2095
2017	123	737	2840
2018	126	743	2572
2019	125	756	2758
2020	58	412	2792
2021	113	613	2689

For each intersecting ADM unit, the geoBoundaries unique identifier is provided (to support joining with the actual ADM unit feature from geoBoundaries if needed), along with the centroid of the ADM feature. In addition, for each ADM unit recorded, we specify an “equal split ratio” representing how many ADM units the project spans in total, as well as the “intersection split ratio” representing the proportion of the project’s geospatial feature which intersects with the ADM unit. For example, a project that intersects two ADM1 units would have an equal split ratio of 0.5 for both ADM units, while the intersection split ratio may be 0.8 and 0.2 if the project resides primarily in one of the ADM units.

Due to imperfect geometry alignment between project geospatial features retrieved from OSM and administrative units defined in geoBoundaries, there are often edge-cases in which very small amounts of overlap occur that do not reflect meaningful project distributions. To minimize the associated noise, we drop all ADM units with intersection ratios under 0.01. The ADM1 and ADM2 level products are included as part of the project-level GCDF v3, and can also be accessed directly via the Geospatial Global Chinese Development Finance Dataset GitHub repository.

#### Incorporating covariate data

To support a broader range of analysis, the data from the Geo-GCDF v3^48^ has also been added to AidData’s GeoQuery platform^54^. GeoQuery is a free-to-use web site that allows users to select customizable boundaries (e.g., a set of administrative zones for a country) and disaggregated measurement data (e.g., temperature, precipitation, population) that they would like aggregated to the specified boundaries. The process allows users with no geospatial expertise or computational resources to leverage large amounts of raw geospatial data in a practical and accessible format. The resulting output is a simple spreadsheet file where each row represents a single unit or boundary, and each column is a measurement dataset (e.g., mean precipitation in 2020).

The Geo-GCDF v3 has been added to GeoQuery as both a boundary dataset and a measurement dataset. Adding the Geo-GCDF v3 as a boundary dataset allows users to extract a range of measurement data to project boundaries, as well as to project boundaries which have been buffered at several intervals (e.g., 1 km). Using these boundaries in GeoQuery, users could, for example, evaluate changes in land cover around project sites contained in the Geo-GCDF v3. The Geo-GCDF v3 has been added as a measurement dataset based on the commitment values of the projects. Using this measurement dataset allows users to select boundaries, such as provinces in Laos, and extract the total commitment amounts associated with projects from the Geo-GCDF v3 in each province.

## Data Availability

All code used to access and process the data involved in the Geo-GCDF v3 is available in the GitHub repository. The repository also contains environment files which define the Python packages and associated versions used, as well as a configuration file that defines specific parameters used. The code is publicly available and free to use with appropriate attribution (see *Citing the Dataset* in the *Usage Notes* section).
