# Author Correction: AidData’s Geospatial Global Chinese Development Finance Dataset

**DOI:** 10.1038/s41597-024-03712-3

**Published:** 2024-09-03

**Authors:** Seth Goodman, Sheng Zhang, Ammar A. Malik, Bradley C. Parks, Jacob Hall

**Affiliations:** AidData, William & Mary, Williamsburg, Virginia USA

**Keywords:** Developing world, Geography

Correction to: *Scientific Data* 10.1038/s41597-024-03341-w, published online 11 June 2024

In this article ref. 20 was incorrect and should have been ‘Ray, R., Gallagher, K. P., Kring, W., Pitts, J. & Simmons, B. A. Geolocated dataset of Chinese overseas development finance. *Sci Data* **8**, 241, 10.1038/s41597-021-01021-7 (2021).

